# Thermal Layout Analysis and Design of Direct Methanol Fuel Cells on PCB Based on Novel Particle Swarm Optimization

**DOI:** 10.3390/mi10100641

**Published:** 2019-09-24

**Authors:** Zhenyu Yuan, Wenhui Chuai, Zhongming Guo, Zhaoyin Tu, Fanbo Kong

**Affiliations:** College of Information Science and Engineering, Northeastern University, Shenyang 110819, China; chuaiwenhuiyc@126.com (W.C.); guo_zhongming@126.com (Z.G.); tuzhaoyin@126.com (Z.T.); K1585817556@126.com (F.K.)

**Keywords:** fuel cells, thermal layout optimization, particle swarm optimization, charge transfer coefficient

## Abstract

As a new energy technology, the fuel cell has developed rapidly, and its performance has been continuously improved. Fuel cell stacks composed of multiple single cells are gradually being used in portable electronic products. Since the performance of fuel cells cannot be optimal at room temperature, it is critical to research cell temperature characteristics and heat distributions in applications. In this paper, the effects of temperature and charge transfer coefficient and the relationship between exchange current density and output voltage were analyzed by the mathematical model of direct methanol fuel cells. Moreover, to optimize the thermal layout of the fuel cell stack in the printed circuit board (PCB) substrate, the idea of a fuel cell as a device was proposed innovatively, and the corresponding thermal optimization strategy was analyzed. A novel particle swarm optimization algorithm was used to detect the optimal layout of fuel cells of different specifications on the same substrate. The three-dimensional thermal simulation model was used to obtain the temperature data and verify the optimization results.

## 1. Introduction

Fuel cells are electrochemical power generation devices that convert the chemical energy of fuel into electric energy. It is one of the potential new energy technologies with the advantages of high energy density, cleanliness and environmental protection, self-generation, and high efficiency, and it is not limited by the Carnot cycle limitation [1,2,3]. Direct methanol fuel cells (DMFCs) use methanol as the reactant, which has the advantage of abundant fuel sources, low price, convenient operation, and easy miniaturization. It is suitable as a power source for portable devices [4,5].

Although DMFC has many advantages, its output power density is much lower than theoretical calculations. Many of studies have found that temperature affects the mass and momentum transfer of DMFC, the methanol concentration, crossover current density, polarization curve, and CO_2_ distribution, etc. [6,7]. A mathematical model for the transient temperature distribution of direct methanol fuel cell (DMFC) was established by Ramesh et al. [8]. Wang et al. added hydrogen peroxide as an oxygen promoter to anodic methanol fuel to improve the rate of methanol electrooxidation in low-temperature direct methanol fuel cells [9]. The results reveal that the optimal temperature operating point of DMFCs is higher than room temperature within a certain range, and temperatures too high or too low will reduce the output performance of DMFCs [10]. Therefore, methods of improving the output power density of DMFCs through temperature has become a key issue. Fang et al. [11] used the electronic control unit and methanol catalytic combustion to make the DMFC work at the optimal operating temperature to improve the output performance of the DMFC. Gregory et al. [12] developed and tested a new steam feeding fuel delivery system for passive DMFCs. The results show that the steam feeding system achieved greater power density than a liquid feeding system for the same DMFC, and its maximum power density is 33 mW/cm^2^ at a current density of 50 mA/cm^2^. Park et al. [13] tested the output performance of a 10-cell methanol fuel stack at −5 °C and −10 °C. The results show that due to the self-heating method of the stack, the stable performance can be properly maintained at a low temperature by setting a reasonable cell switch and operating mode. However, all additional equipment is required for implementation, increasing the complexity of the fuel cell power supply system, and thus resulting in a limited net power and energy efficiency.

With the rapid development of the electronic industry and micro-electromechanical system (MEMS) technology, micro fuel cells are gradually being integrated into portable electronic products. Many studies have explored the application of printed circuit board (PCB) technology in fuel cell manufacturing and analyzed its performance. O’Hayre et al. [14] used PCB technology to fabricate 16-cell portable oxyhydrogen fuel cells, demonstrating that the combination of fuel cells and PCB technology has the potential to improve power density and produces promising synergies, such as adding electronic devices and programmable automation control. Aricò et al. [15] investigated the performance of a PCB-integrated three-cell DMFC stack at different concentrations and catalyst loadings. The results show that the maximum power was 225 mW with a corresponding power density of 20 mW/cm^2^. Baglio et al. [16] compared the performance of PCB-integrated three-cell DMFC stacks with different current collectors. The stack with a large aperture current collector and the stack with a small aperture current collector achieved similar maximum power density. The stack with a large aperture current collector achieved better mass transfer characteristics and had a longer discharge time. The stack with a small aperture current collector had smaller methanol permeability at low current. Kuan et al. [17] referred to the planar PCB-DMFC module and analyzed the performance of DMFCs made from Hilbert curve fractal plates. The results show that the larger the opening ratio and the opening perimeter, the higher the performance. Yuan et al. [18] fabricated an eight-cell mono-polar DMFC stack with PCB technology. The feasibility of constructing an eight-cell mono-polar DMFC stack based on PCB technology was verified by experiments. In the above studies, the fuel cells test-fabricated by PCB technology was conducted by analyzing the influence of parameters on fuel cell performance. The advantages of PCB technology in reducing the design weight of DMFCs and its potential application in the production of collectors or flow distributors were highlighted. However, the heat-releasing capacity of DMFCs and the influence of plate layout on fuel cell performance have not been considered.

The components in the circuit board will emit heat during the operation, and the temperature of each component on the PCB will affect each other, which has an impact on the performance of the entire circuit. Therefore, the layout of the electronic devices is a key issue. Meanwhile, the innovative concept—DMFC as the component in the circuit board—is introduced in our design. For DMFCs in which output performance is greatly affected by temperature, whether DMFC is applied to the circuit board as an electronic component or the DMFC stack is cascaded through the PCB, and the temperature of the DMFC is affected by the layout. This will adversely affect the output performance of the fuel cells. Most devices generate a large amount of heat in the circuit board, so the output performance of the DMFC can only be improved by reasonable device layout, without external heating.

Particle Swarm Optimization (PSO) is an evolutionary computation method [19]. Inspired by the law of bird’s swarm activity, this algorithm uses swarm intelligence to establish a simplified model. PSO makes use of information sharing between individuals to make the movement of the whole population evolve from disorder to order in the solution space so as to obtain the optimal solution. In recent years, PSO has been widely used to solve optimization problems because of its simple algorithm, fast search speed, and high efficiency [20]. Based on the above understanding, we propose a particle swarm optimization algorithm to optimize the thermal layout of DMFCs and electronic components. Firstly, a mathematical model was established for the heat transfer effect of DMFCs. Furthermore, the total heat flux of each DMFC calculated by the mathematical model was used as the heat source. The thermal distribution of DMFCs and other devices on the substrate was simulated by COMSOL Multiphysics. The fitness function was established based on the thermal simulation data, and the particle swarm optimization algorithm was used to detect the optimal layout of the DMFC. Finally, the optimized layout coordinates were substituted into the substrate for simulation verification. The article provides theoretical basis and application reference for the application of fuel cells for integrated circuits.

## 2. DMFC Model

**Figure 1:**
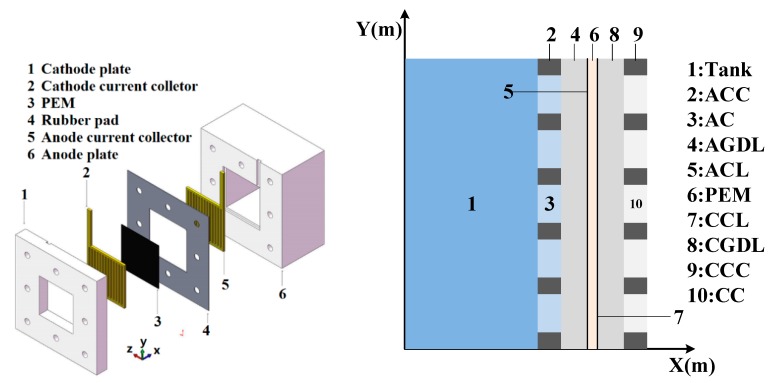
Simplified structure diagram of passive direct methanol fuel cell (DMFC).

This model was established on the basis of our previous study and the heat transfer model [21]. In order to simplify the calculation, we made the following simplifications and assumptions. The DMFC operates under steady-state conditions. The methanol concentration in the reservoir cavity is assumed to be constant. It was assumed that the anode tank, anode current collector (ACC), and anode gas diffusion layer (AGDL) are both well insulated from the environment. Heat transfer and mass transfer in the diffusion layer are both dominant in the diffusion process. It was assumed that the methanol in the cathode catalyst layer (CCL) is completely removed by reaction. The simplified structure of the DMFC is shown in Figure 1. In this figure, the left figure is the three-dimensional structure of the fuel cell, and the right figure is the schematic diagram of the X-Y two-dimensional coordinates, with the X and Y coordinates representing the size of the DMFC.

### 2.1. Mass Transfer Model

The internal mass transfer process of DMFCs can be divided into two regions: anode and cathode. At the anode, the methanol solution is transferred from the tank, ACC, AGDL, and sequentially to the anode catalyst layer (ACL), and part passes through the proton exchange membrane (PEM) to the cathode to react with oxygen. At the cathode, oxygen is sequentially transferred from the air, the cathode current collector (CCC), and the cathode gas diffusion layer (CGDL) to the CCL and partially reacts with the methanol crossover.

According to convective mass transfer characteristics and Fick’s law, the mass transfer process of methanol from the tank to the ACL can be expressed by the following equations:

(1) $$\{\begin{array}{c} N_{m}=h_{m}\left(C_{m,tank}-C_{m,acc}^{0}\right)\\ N_{m}=-D_{m,acc}^{eff}\frac{dC_{m,acc}}{dx}\\ N_{m}=-D_{m,agdl}^{eff}\frac{dC_{m,agdl}}{dx} \end{array}\Rightarrow \{\begin{array}{c} N_{m}=\alpha_{1}\left(C_{m,tank}-C_{m,acl}\right)\\ \alpha_{1}={\left(\frac{l_{agdl}}{D_{m,agdl}^{eff}}+\frac{l_{acc}}{D_{m,acc}^{eff}}+\frac{1}{h_{m}}\right)}^{-1} \end{array}$$

where $N_{m}$ represents the flux of methanol, $h_{m}$ represents the mass transfer coefficient on the surface of the anode plate, and $C_{m,acc}^{0}$ and $C_{m,\tan k}$ represent the concentration of methanol solution on the surface of the anode plate and in the anode reservoir cavity, respectively. $D_{m,acc}^{eff}$ and $D_{m,agdl}^{eff}$ represent the effective diffusion coefficients of methanol in the anode plate and the anode diffusion layer, respectively.

The methanol flux on the proton exchange membrane is related to the proton current density. Considering the methanol crossover from the PEM to the cathode, the anode overpotential is solved by Tafel’s law and Fick’s law, and the following equations are established:

(2) $$\{\begin{array}{c} N_{m}=\frac{1}{6F}i_{a}+N_{cross};i_{a}=i_{ref}^{m}\frac{C_{m,acl}}{C_{ref}^{m}}exp\left(\frac{\alpha_{a}F}{RT_{acl}}\eta_{a}\right)\\ N_{cross}=-D_{m,mem}^{eff}\frac{dC_{m,mem}}{dx}+n_{d}^{m}\frac{i}{F} \end{array}\Rightarrow \eta_{a}=\frac{RT_{acl}}{\alpha_{a}F}ln\left(\frac{iC_{ref}^{m}}{\delta_{acl}j_{a}C_{m,acl}}\right)$$

where $i_{a}$ is the proton current density, $N_{cross}$ represents the permeation flux of methanol through the proton exchange membrane, $D_{m,mem}^{ref}$ is the effective diffusion coefficient of methanol in the proton exchange membrane, $n_{d}^{m}$ is the electro-osmotic resistance coefficient of methanol, $\delta_{acl}$ is the thickness of the anode catalyst layer, and $j_{a}$ is the unit volume anode reference exchange current density.

According to convective mass transfer characteristics and Fick’s law, the mass transfer process of oxygen from the air to the cathode catalytic layer can be expressed by the following equation:

(3) $$\{\begin{array}{c} N_{O_{2}}=h_{O_{2}}\left(C_{O_{2},amb}-C_{O_{2},ccc}^{0}\right)\\ N_{O_{2}}=-D_{O_{2},cgdl}^{ref}\frac{dC_{O_{2},cgdl}}{dx};N_{O_{2}}=-D_{O_{2},ccc}^{ref}\frac{dC_{O_{2},ccc}}{dx} \end{array}\Rightarrow \{\begin{array}{c} N_{O_{2}}=\alpha_{2}\left(C_{O_{2},amb}-C_{O_{2},ccl}\right)\\ \alpha_{2}={\left(\frac{l_{cgdl}}{D_{O_{2},cgdl}^{eff}}+\frac{l_{ccc}}{D_{O_{2},ccc}^{eff}}+\frac{1}{h_{O_{2}}}\right)}^{-1} \end{array}$$

At the cathode, a portion of the oxygen reacts with the crossover methanol solution to generate an internal current and a mixed potential. Considering the internal current, Tafel’s law is used to solve the cathode overpotential, and the following equation is established:

(4) $$\{\begin{array}{c} N_{O_{2}}=\frac{1}{4F}i_{c}+\frac{3}{2}N_{cross};i_{p}=6FN_{cross}\\ i_{c}+i_{p}=i_{ref}^{O_{2}}\frac{C_{O_{2},ccl}}{C_{ref}^{O_{2}}}exp\left(\frac{\alpha_{c}F}{RT_{ccl}}\eta_{c}\right) \end{array}\Rightarrow \;\eta_{c}=\frac{RT_{ccl}}{\alpha_{c}F}ln\left(\frac{iC_{ref}^{O_{2}}}{\delta_{ccl}j_{c}C_{O_{2},ccl}}\right)$$

### 2.2. Performance Model

According to the polarization curves of passive DMFC, the output voltage of DMFC can be expressed as:

(5) $$V_{cell}=E_{cell}-\eta_{a}-\eta_{c}-iR_{cell}$$

where $E_{cell}$ is the thermodynamic equilibrium potential of the fuel cell and is a function of temperature and pressure. $R_{cell}$ is the internal resistance of the fuel cell.

The thermodynamic equilibrium potential of fuel cells can be calculated as:

(6) $$E_{cell}=E_{cell}^{0}+\left(T_{acl}-T_{amb}\right)\frac{\partial E}{\partial T}$$

where $E_{cell}^{0}$ is the open circuit voltage at T=298 K, and $\frac{\partial E}{\partial T}$ represents the rate of change of the electromotive force.

### 2.3. Heat Transfer Model

The heat generated by the electrochemical reaction of ACL can be expressed as

(7) $$q_{acl}=i\left(\eta_{a}-\frac{\Delta H_{a}-\Delta G_{a}}{nF}\right)$$

The first item represents the heat generated by the anode activation and overpotential of the mass transfer. The second one is the entropy change of anode electrochemical reaction.

Neglecting the Joule heat generated in the PEM, the heat flux across the PEM can be expressed as

(8) $$q_{acl}=-\lambda_{mem}\frac{dT}{dx}$$

where $\lambda_{mem}$ is the effective thermal conductivity of the proton exchange membrane.

Considering the effects of crossover current and vaporization of liquid water, the heat generated in the CCL can be expressed as

(9) $$q_{ccl}=\left(i+i_{p}\right)\eta_{c}-i\frac{\Delta H_{c}-\Delta G_{c}}{nF}-h_{v}N_{H_{2}O}$$

The first item is the heat generated by activation, mass transfer overpotential, and the mixed potential generated by methanol crossover; the second item represents the loss of entropy; and the third one is the heat generated by gasification of liquid water in the CCL.

The flux of water evaporation is expressed by natural convection as

(10) $$\{\begin{array}{c} N_{H_{2}O}=h_{H_{2}O}\left(C_{H_{2}O,ccc}^{sat}-C_{H_{2}O,amb}\right);C_{H_{2}O,ccc}^{sat}=\frac{P_{H_{2}O}}{RT_{ccc}}\\ P_{H_{2}O}=10^{5}\times 10^{\left(\begin{array}{c} -2.1794+0.02953\times T-9.1837\\ \times 10^{-5}\times T^{2}+1.4454\times 10^{-7}\times T^{3} \end{array}\right)}\\ T=T_{ccc}-273 \end{array}$$

where $h_{H_{2}O}$ represents the mass transfer coefficient of water vapor at the cathode, and $C_{H_{2}O,CCC}^{sat}$ and $C_{H_{2}O,amb}$ represent the concentration of water vapor at the cathode plate surface and in the air, respectively. The concentration of water vapor can be expressed as $\frac{P_{H_{2}O}}{RT}$, and the saturated pressure in humid air can be determined.

The total heat includes the heat produced by the ACL and the CCL. The total heat is gradually transferred to the CGDL, the cathode channel (CC), and the air. The heat transfer can be expressed by the following equation:

(11) $$\{\begin{array}{c} q_{tot}=q_{acl}+q_{ccl}\\ q_{tot}=-\lambda_{cgdl}\frac{dT}{dx}\\ q_{tot}=-\lambda_{ccc}\frac{dT}{dx}\\ q_{tot}=h_{t}\left(T_{ccc}^{0}-T_{amb}\right) \end{array}\Rightarrow \{\begin{array}{c} q_{tot}=\alpha_{3}\left(T_{ccl}-T_{amb}\right)\\ \alpha_{3}={\left(\frac{1}{h_{t}}+\frac{l_{cgdl}}{\lambda_{cgdl}}+\frac{l_{ccc}}{\lambda_{ccc}}\right)}^{-1} \end{array}$$

where $\lambda_{cgdl}$ and $\lambda_{ccc}$ represent the effective thermal conductivity of the cathode diffusion layer and the cathode plate, respectively; $h_{t}$ represents the tropospheric heat transfer coefficient of natural convection; and $T_{ccc}^{0}$ and $T_{amb}$ represent the temperature at the cathode plate surface and the ambient temperature, respectively.

The natural convection heat transfer coefficient on the CCC surface can be expressed as

(12) $$\{\begin{array}{c} h_{t}=\frac{\lambda Nu}{L};Nu=0.68+\frac{0.67Ra_{L}^{1/4}}{{\left[1+{\left(\frac{0.492}{Pr}\right)}^{6/16}\right]}^{4/9}}\\ Ra_{L}=\frac{\beta g\left(T_{CCC}-T_{amb}\right)L^{3}}{\mu^{2}}Pr \end{array}$$

The convection mass transfer coefficient on the anode and cathode can be expressed as

(13) $$\{\begin{array}{c} h=\frac{Le\cdot Nu\cdot D}{L};Nu=0.68+\frac{0.67Ra_{L}^{1/4}}{{\left[1+{\left(\frac{0.492}{Pr}\right)}^{6/16}\right]}^{4/9}} \end{array}$$

where Nu represents Nussel number, $Ra_{L}$ is the Rayleigh number, μ is the Kinematic viscosity, Pr is the Plante number, and D is the diffusion coefficient. Le is the Lewis number; for gas, Le=1, and for liquids, Le=2.

### 2.4. Effect of Temperature on Output Performance

The charge transfer coefficient (CTC) is one of the key parameters affecting the cell performance, and it is also an important parameter affected by temperature in the electrochemical reaction [22]. Typically, the CTC value is obtained from polarization curve and the Tafel slope [23]. The anode and cathode transfer coefficients in electrochemistry are greatly affected by temperature, which can be expressed as

(14) $$\mathrm{Anodic}\;\mathrm{CTC}:\;\frac{\alpha_{a}}{\nu }=\left(\frac{RT}{nF}\right)\left(\frac{\partial ln\left(\left|I_{ox}\right|\right)}{\partial E}\right)$$

(15) $$\mathrm{Cathodic}\;\mathrm{CTC}:\;\frac{\alpha_{c}}{\nu }=-\left(\frac{RT}{nF}\right)\left(\frac{\partial ln\left(\left|I_{red}\right|\right)}{\partial E}\right)$$

where ν is the stoichiometric number, *n* is the number of electrons in the electrode reaction, *E* is the electrode potential, $I_{ox}$ is the current of anodic oxidation, and $I_{red}$ is the current of cathodic reduction. The physical parameters used in the model are listed in Table 1.

**Figure 2:**
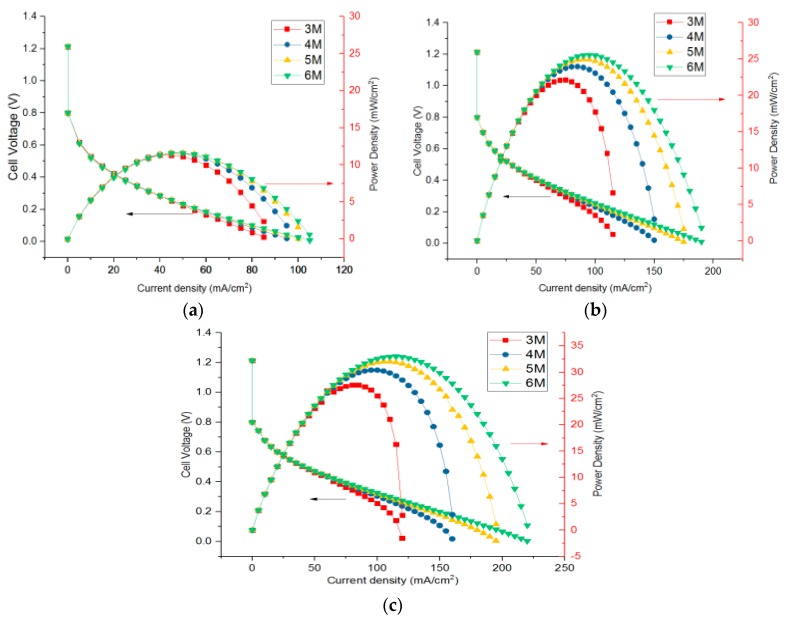
Output performance of the DMFC at different charge transfer coefficients (CTCs). (**a**) $\alpha_{a}$ is 0.35 and $\alpha_{c}$ is 0.8. (**b**) $\alpha_{a}$ is 0.45 and $\alpha_{c}$ is 0.85. (**c**) $\alpha_{a}$ is 0.5 and $\alpha_{c}$ is 0.875.

**Figure 3:**
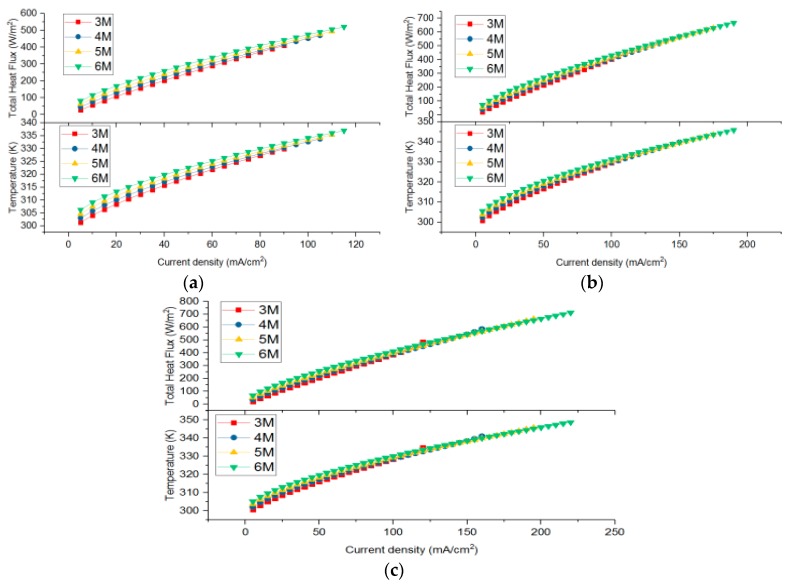
Heat flux and temperature of the cathode current collector (CCC) at different CTCs. (**a**) is 0.35 and $\alpha_{c}$ is 0.8. (**b**) $\alpha_{a}$ is 0.45 and $\alpha_{c}$ is 0.85. (**c**) $\alpha_{a}$ is 0.5 and $\alpha_{c}$ is 0.875.

It can be seen from the characteristics of the transfer coefficient that, within a certain range, the transfer coefficient increases with the increase of the temperature. In this paper, typical transfer coefficients were selected as shown in Table 2, and the established model was used to solve the I–V and I–P curve, as shown in Figure 2 and Figure 3.

It can be seen from Figure 2 that within a certain range, the activation overpotential decreases with the increase of the transfer coefficient, the maximum power density increases, and the output voltage can be stabilized at a higher current density. For example, the maximum power density increased from 11.41 mA/cm^2^ to 27.54 mA/cm^2^ when the concentration of methanol was 3 M. In the process of changing the concentration from 3 M to 6 M, the performance increased gradually, but the increment became smaller. When the anode CTC was 0.45 as shown in Figure 2b, the maximum power density was 22.12 mW/cm^2^, 23.96 mW/cm^2^, 24.98 mW/cm^2^, and 25.54 mW/cm^2^.

As seen from Figure 3, with the increase of current density, the temperature and heat flux of CCC showed the same upward trend, but the growth rate slowed down. When the CTC was relatively high, the temperature and heat flux were small at the same current density. Temperature and heat flux increased when methanol concentration increased from 3 M to 6 M. As shown in Figure 2b and Figure 3b, at the maximum power density, the corresponding temperatures of the CCC were 311.42 K, 326.48 K, 328.39 K, and 330.26 K. Meanwhile, as the CTC increased, the concentration polarization of the DMFC became more obvious at a low concentration, and the temperature of the CCC increased rapidly at concentration polarization.

The influence of temperature on the output performance of the fuel cell is not only reflected in the charge transfer coefficient but also affects the exchange current density, internal resistance, flooding, and proton exchange membrane performance, which is a complicated process with several factors. In order to analyze the effect of temperature on the performance of DMFC, the relationship between charge transfer coefficient and unit volume reference exchange current density and temperature was considered based on the above model. Both the exchange current density and the CTC increased with the increase of temperature. The relationship between the established charge transfer coefficient and the reference volume exchange current density per unit volume is as follows:

(16) $$\{\begin{array}{c} j_{a}=11\times 10^{6}\times \exp\left(-\frac{10^{3}}{T_{acc}}-\frac{10^{3}}{T_{amb}-25}\right)\\ j_{c}=11\times 10^{6}\times \exp\left(-\frac{10^{3}}{T_{ccc}}-\frac{10^{3}}{T_{amb}-25}\right)\\ \alpha_{a}=0.35+0.2\times \left(1-\frac{1}{2^{\frac{T_{amb}-298}{15}}}\right)\\ \alpha_{c}=0.8+0.1\times \left(1-\frac{1}{2^{\frac{T_{amb}-298}{15}}}\right) \end{array}$$

**Figure 4:**
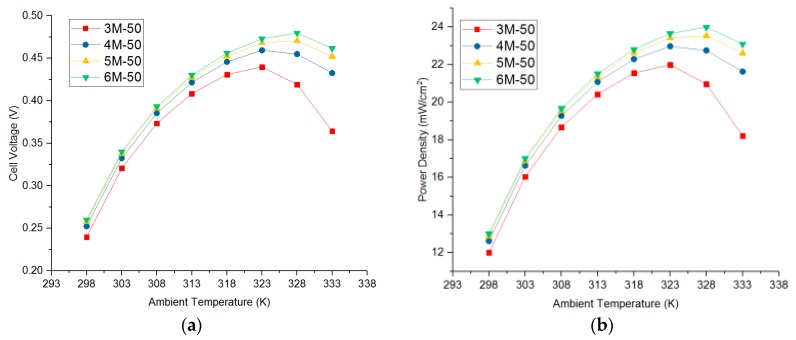

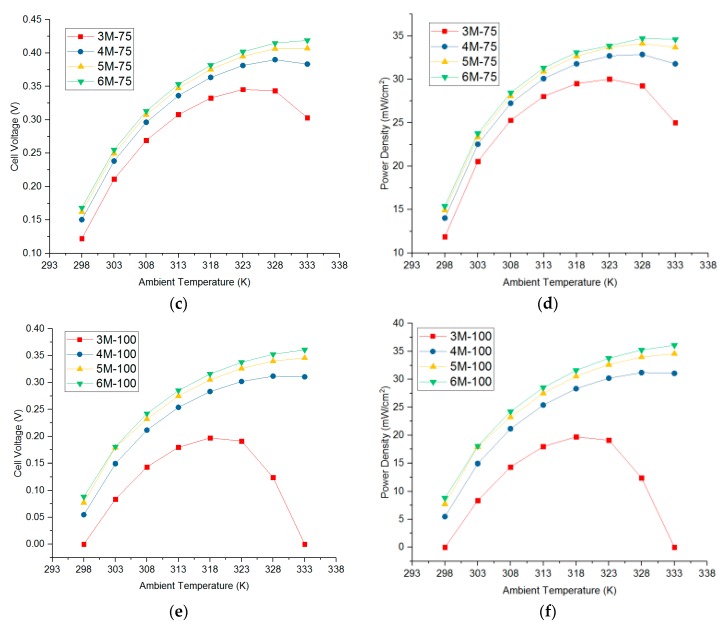
Relationship between output voltage (OV) and power density (PD) and ambient temperature (AT) under different current densities. (**a**) Relationship between AT and OV at 50 mA/cm^2^. (**b**) Relationship between AT and PD at 50 mA/cm^2^. (**c**) Relationship between AT and OV at 75 mA/cm^2^. (**d**) Relationship between AT and PD at 75 mA/cm^2^. (**e**) Relationship between AT and OV at 100 mA/cm^2^. (**f**) Relationship between AT and PD at 100 mA/cm^2^.

According to the model considering the influence of ambient temperature, the relationship between temperature and output performance was obtained at different current densities. It can be seen from Figure 4 that the output voltage and power density increase greatly within a certain range as the ambient temperature rises. When the temperature is too high, the output performance drops sharply, which is mainly caused by the increase of the CTC and the gradual decrease of the exchange current density. When the concentration was 3 M at a current density of 100 mA/cm^2^, the output voltage and power density were zero at 298 K and 333 K, which is mainly due to the current density exceeding the output range (the unit K appearing in the text represents the Kelvin temperature). When the concentration increased from 3 M to 6 M, the performance was improved at high temperatures; but with the increase of concentration, the performance improved less. The optimum ambient temperature of the DMFC was different at different current densities. For example, when the concentration was 6 M and the current density was 50 mA/cm^2^, the optimum operating temperature was 328 K; when the concentration was 6 M and the current density was 75 mA/cm^2^, the optimum operating temperature was higher than 333 K. As shown in Figure 4b, the maximum power densities of 3–6 M were 22.00 mW/cm^2^, 22.97 mW/cm^2^, 23.53 mW/cm^2^, and 23.99 mW/cm^2^, respectively. As shown in Figure 4d, the highest power densities were 25.91 mW/cm^2^, 29.26 mW/cm^2^, 30.53 mW/cm^2^, and 31.43 mW/cm^2^. As shown in Figure 4f, the highest power densities were 19.71 mW/cm^2^, 31.20 mW/cm^2^, 34.58 mW/cm^2^, and 36.09 mW/cm^2^.

## 3. Establishment and Simulation of Heat Distribution Model

It can be seen from the analysis of the above mathematical model that the increase of the temperature within a certain range can improve the reaction characteristics inside the DMFC and greatly improve the performance of the DMFC. However, the optimum operating temperature of a single DMFC is higher than room temperature, and the ambient temperature needs to be raised to achieve better output performance. Detailed DMFC preparation, temperature performance testing, and thermal optimization methods have been described in our previous paper [26]; based on previous experimental test results and potential portable applications of fuel cells, this paper proposes a method that using PSO to calculate the optimal layout of bipolar plates or some single-cells to improve the ambient temperature of each DMFC. This method can improve the performance of DMFCs by heating without additional heating equipment.

The thermal layout model was built using COMSOL Multiphysics to analyze the heat transfer of multiple DMFC layouts. The size of the PCB was 0.1 m × 0.1 m. DMFCs and heating devices were simplified to squares. The thickness of the plate and the thickness of the device were both 10^−3^ m, and the dimensional accuracy was 10^−4^ m. The physical parameters are shown in Table 3. The total heat flux of DMFCs with different sizes was calculated according to the mathematical model of DMFCs. The total heat flux serves as the heat source of each cell. The three-dimensional solid heat transfer model was built by using simulation software to simulate the layout of the device on the substrate, and the heat sources under different conditions are shown in Table 4.

## 4. PSO for Optimal Layout

Due to the heat transfer, the different layouts of the DMFC have an influence on the temperature of each DMFC itself. In this paper, C++ language and PSO were used to detect the optimal layout coordinates. It is necessary to set parameters related to the algorithm and establish a fitness function in the process of solving the PSO algorithm.

### 4.1. PSO Algorithm Formula and Parameter Setting

In this paper, the inertia weight and the speed of PSO were improved, with the main formula shown in Equations (17) and (18).

The speed update Equation of PSO is

(17) $$Vi_{t+1}=wVi_{t}+c_{1}\cdot r_{1}\left(Pi_{t}-Zi_{t}\right)+c_{2}\cdot r_{2}\left(Pg_{t}-Zi_{t}\right)$$

The displacement update Equation of PSO algorithm is

(18) $$Zi_{t+1}=Zi_{t}+Vi_{t+1}$$

where $Vi_{t}$ and $Vi_{t+1}$ represent the velocity of particle *i* at *t* and *t* + 1 iterations, respectively; *w* is inertial weight, which can change the search range and search speed of particles; $c_{1}$ and $c_{2}$ are learning factors; $r_{1}$ and $r_{2}$ are random numbers in the interval of [0,1]; $Pi_{t}$ is the optimal value experienced by particle *i* in the *t* iteration; $Pg_{t}$ is the optimal value experienced by particle swarm in the *t* iteration; $Zi_{t}$ and $Zi_{t+1}$ is the position of the particle *i* at the *t* and *t* + 1 iterations, respectively; and *Z* can represent both the X-coordinate and the Y-coordinate.

The setting of *w* is based on the combination of linear decrement and periodic adjustment, which can better search for the optimal location.

(19) $$w=\{\begin{array}{c} 2-\left(\frac{1.7t}{N-1}\right)\;\;\;\;\;\;\;\;\;\;\;\;\;t\left(mod5\right)\neq 0\\ 0.8\;\;\;\;\;\;\;\;\;\;\;\;\;\;\;\;\;\;\;\;\;\;\;\;\;\;\;t\left(mod5\right)=0 \end{array}$$

The update velocity is limited, which is halved beyond the scope until the restriction of (20) is achieved.

(20) −0.001<V<0.001

### 4.2. Establishment of Fitness Function

The fitness function is the basis of the optimal layout by PSO. In this paper, a thermal simulation model was used to obtain the data between the temperature and the coordinate, and the fitness function of each device was established. The layouts of different sizes were studied, in which the ambient temperature was 298 K, the concentration was 3 M, and the current density was 50 mA/cm^2^. Situation 1: The dimensions are all 1.0 cm. Situation 2: The dimensions are all 1.0 cm, but one of the heat sources is set to 600 W/m^2^. Situation 3: The dimensions of four device are different, with the values of 2.0 cm, 1.5 cm, 1.0 cm and 0.5 cm, respectively. The heat sources corresponding to different sizes are set according to Table 5, and the distance between each device layout is less than 0.1 cm. Different fitness functions were established according to different situations, as shown in Equations (21)–(23).

Situation 1:

(21) $$\{\begin{array}{ll} T1= & 306+\frac{3.4}{{\left(x12+y12-10\right)}^{0.7}}+\frac{3.4}{{\left(x13+y13-10\right)}^{0.7}}\\ & +\frac{3.4}{{\left(x14+y14-10\right)}^{0.7}}-10\left(\left|x1\right|+\left|y1\right|\right)\\ T2= & 306+\frac{3.4}{{\left(x12+y12-10\right)}^{0.7}}+\frac{3.4}{{\left(x23+y23-10\right)}^{0.7}}\\ & +\frac{3.4}{{\left(x24+y24-10\right)}^{0.7}}-10\left(\left|x2\right|+\left|y2\right|\right)\\ T3= & 306+\frac{3.4}{{\left(x13+y13-10\right)}^{0.7}}+\frac{3.4}{{\left(x23+y23-10\right)}^{0.7}}\\ & +\frac{3.4}{{\left(x34+y34-10\right)}^{0.7}}-10\left(\left|x3\right|+\left|y3\right|\right)\\ T4= & 306+\frac{3.4}{{\left(x14+y14-10\right)}^{0.7}}+\frac{3.4}{{\left(x24+y24-10\right)}^{0.7}}\\ & +\frac{3.4}{{\left(x34+y34-10\right)}^{0.7}}-10\left(\left|x4\right|+\left|y4\right|\right) \end{array}$$

Situation 2:

(22) $$\{\begin{array}{ll} T1= & 320+\frac{5.4}{{\left(x12+y12+x13+y13+x14+y14-10\right)}^{0.5}}\\ & -10\left(\left|x1\right|+\left|y1\right|\right)\\ T2= & 306+\frac{5.8}{{\left(x12+y12-10\right)}^{0.7}}+\frac{4.2}{{\left(x23+y23-10\right)}^{0.7}}\\ & +\frac{4.2}{{\left(x24+y24-10\right)}^{0.7}}-10\left(\left|x2\right|+\left|y2\right|\right)\\ T3= & 306+\frac{5.8}{{\left(x13+y13-10\right)}^{0.7}}+\frac{4.2}{{\left(x23+y23-10\right)}^{0.7}}\\ & +\frac{4.2}{{\left(x34+y34-10\right)}^{0.7}}-10\left(\left|x3\right|+\left|y3\right|\right)\\ T4= & 306+\frac{5.8}{{\left(x14+y14-10\right)}^{0.7}}+\frac{4.2}{{\left(x24+y24-10\right)}^{0.7}}\\ & +\frac{4.2}{{\left(x34+y34-10\right)}^{0.7}}-10\left(\left|x4\right|+\left|y4\right|\right) \end{array}$$

Situation 3:

(23) $$\{\begin{array}{ll} T1= & 314.5+\frac{1.5}{x13+y13-15}+\frac{8}{x12+y12-17.5}\\ & -10\left(\left|x1\right|+\left|y1\right|\right)\\ T2= & 328+\frac{3}{x12+y12-17.5}+\frac{0.5}{x23+y23-12.5}\\ & -0.5e^{\left(1-10\left|x1\right|\right)}\\ T3= & 307+\frac{13}{x23+y23-12.5}+\frac{5}{x13+y13-15}\\ & -10\left(\left|x3\right|+\left|y3\right|\right)\\ T4= & 302+\frac{14}{{\left(x14+y14-12.5\right)}^{2}}+\frac{19}{{\left(x24+y24-10\right)}^{2}}\\ & -10\left(\left|x4\right|+\left|y4\right|\right)-\frac{40}{{\left(x14\;+\;y14\;+\;x24\;+\;y24\;-\;22.5\right)}^{2}} \end{array}$$

where x1, y1, x2, y2, x3, y3, x4, y4 are the abscissas and ordinates of the four devices; x12, y12, x13, y13, x14, y14, x23, y23, x24, y24, x34, y34 are 1000 times the distance between the coordinates of each device. In addition, we take the location of the geometric center as the coordinates of each device.

### 4.3. Process of Searching for Optimal Layout

PSO was used to detect the optimal layout. Firstly, the dimensions of heating devices, fuel cells and circuit boards were determined. Secondly, fuel cells and heating devices were regarded as particles to form a particle swarm, and the temperature-coordinate relationship was constructed as a fitness function. Thirdly, the improved PSO was used to search for the optimal device coordinates. The program execution procedure of PSO was realized by the C++ language.

## 5. Simulation Verification

**Figure 5:**
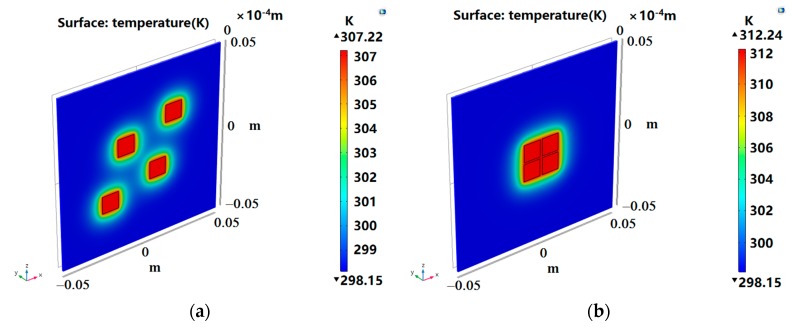
Situation 1: initial layout (**a**) and optimized layout (**b**).

**Figure 6:**
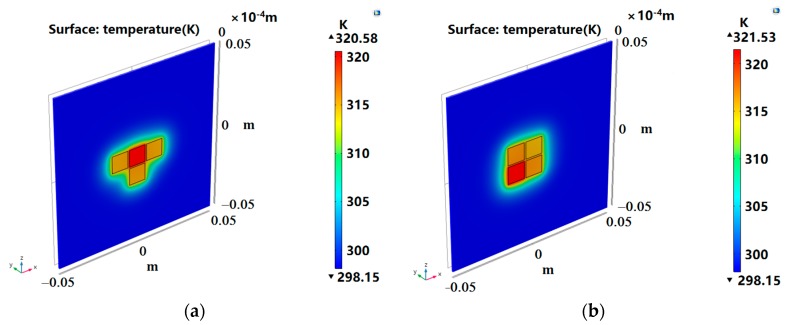
Situation 2: initial layout (**a**) and optimized layout (**b**).

**Figure 7:**
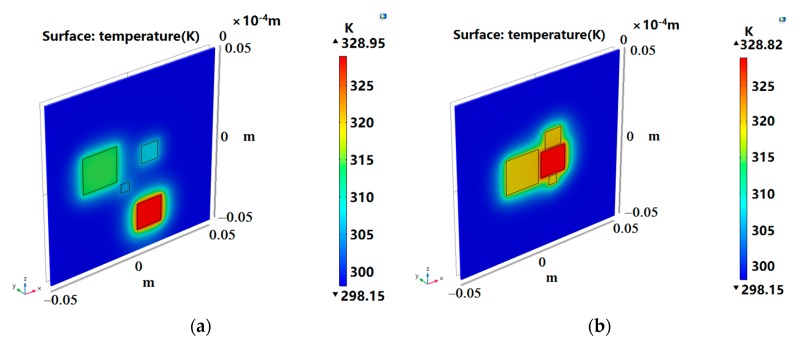
Situation 3: initial layout (**a**) and optimized layout (**b**).

The proposed three distributions are verified in this section. COMSOL Multiphysics was used to establish a thermal layout model to analyze the heat transfer of the DMFC layout. By comparison, the initial layout of the three cases was simulated to obtain the temperature distribution, as shown in Figure 5a, Figure 6a, and Figure 7a. Since the final result did not exceed the optimal operating temperature of the DMFC, we measured the layout with the total temperature of the four devices, with the principle that the higher the total temperature is, the better the layout is. The optimized layout of the above three situations are shown in Figure 5b, Figure 6b, and Figure 7b, respectively.

The temperature calculated by program and the temperature verified by simulation are listed in Table 5. The temperature error of each device was within 1 K, which indicates that the calculation result is consistent with the verification result, and the fitness function meets the optimization requirements. As shown in Table 6, the optimized layout had a significant improvement over the total temperature of the initial layout. Under the three situations, the total temperatures of optimized layout were 19.90 K, 3.84 K, and 32.1 K higher than that of the initial layout, respectively. As can be seen from Figure 4, the increase of ambient temperature made the DMFC exhibit a better performance. For example, in Situation 1, the temperature of the 0.5 cm DMFC rose from about 307 K to 312 K, increasing by about 5 K. As shown in Figure 4b, the power density increased from 18.67 mW/cm^2^ to 20.43 mW/cm^2^ when the ambient temperature rose from 308 K to 313 K. In Situation 3, for example, the temperature of each DMFC rose from about 305 K to 319 K, which was increased by about 14 K. As shown in Figure 4b, the power density increased from 16.03 mW/cm^2^ to 21.55 mW/cm^2^ when the ambient temperature rose from 303 K to 318 K. This shows that it is very effective to improve the output performance by increasing the temperature of the layout. PSO can be used to optimize the placement and improve the overall performance of DMFC.

## 6. Conclusions

In this paper, the mathematical model of DMFC was established based on the reaction principle of DMFC, which considered the relationship between ambient temperature and the charge transfer coefficient. According to the model, the influence of ambient temperature and the charge transfer coefficient on the performance of DMFC was analyzed. The calculated results are in good agreement with our previous experimental results. The correctness of the simulation could be proved [26,27,28]. The power density of DMFC increased gradually when the ambient temperature increased from 298 K to 323 K at the concentration of 3 M; and when the ambient temperature was higher than 323 K, the performance of DMFC decreased. When the concentration increased from 3 M to 6 M, the optimum working temperature increased slightly. We proposed to use PSO to determine the thermal layout and proved that this method can improve the output performance of DMFCs in practical application. The layout method was applied to analyze three cases and better layouts were determined. The temperature of each DMFC was increased, and the total temperature of the optimized layout were 19.90 K, 3.84 K, and 32.10 K higher than the total temperature of the initial layout, respectively, which corresponds to higher output power densities in the mathematical model.

## Figures and Tables

**Table 1:** Physical parameters used in the model.

Symbol (unit)	Parameter	Value
$\Delta H_{m}$ (J/mol)	Enthalpy of formation of liquid methanol	−2.3866 × 10^5^
$\Delta H_{w}$ (kg/mol)	Enthalpy of formation of liquid water	−2.8583 × 10^5^
$\Delta H_{CO_{2}}$ (kg/mol)	Enthalpy of formation of carbon dioxide	−3.9351 × 10^5^
$\Delta G_{m}$ (kg/mol)	Gibbs free energy of liquid methanol	−1.6627 × 10^5^
$\Delta G_{w}$ (kg/mol)	Gibbs free energy of liquid water	−2.3708 × 10^5^
$\Delta G_{CO_{2}}$ (kg/mol)	Gibbs free energy of carbon dioxide	−3.94 × 10^5^
$c_{m}$ (J/(mol·K))	Specific heat capacity of liquid methanol	80.96
$c_{H_{2}O}$ (J/(mol·K))	Specific heat capacity of liquid water	75.24
$c_{CO_{2}}$ (J/(mol·K))	Specific heat capacity of carbon dioxide	36.9
$c_{O_{2}}$ (J/(mol·K))	Specific heat capacity of oxygen	39.44
$h_{v}$ (J/mol)	Latent heat of vaporization of water	4.486 × 10^4^
*F* (C/mol)	Faraday constant	96,458
*R* (J/mol·K)	General gas constant	8.3143
$P_{0}$ (Pa)	Cathode air pressure	1 × 10^5^
$C_{O_{2},amb}$ (mol/m^3^)	Oxygen concentration in the environment	$0.21P_{0}/\left(RT_{amb}\right)$
$l_{acc}$ (m)	Thickness of ACC	0.001
$l_{ccc}$ (m)	Thickness of CCC	0.001
$\;l_{agdl}$ (m)	Thickness of AGDL	0.3 × 10^−3^
$l_{cgdl}$ (m)	Thickness of CGDL	0.3 × 10^−3^
$\delta_{acl}$ (m)	Thickness of ACL	0.5 × 10^−4^
$\delta_{ccl}$ (m)	Thickness of CCL	0.3 × 10^−4^
$l_{mem}$ (m)	Thickness of PEM	0.2 × 10^−3^
*L* (m)	Length of electrode	0.01
$n_{H_{2}O}$	Electro-osmotic drag coefficient of water	2.9exp(1029 × (1/333 − 1/T))
$n_{d}^{m}$	Electro-osmotic drag coefficient of methanol	0.065$n_{H_{2}O}$
ε	Diffusion correction factor	0.6
$\lambda_{mem}$ (W/(m·K))	Thermal conductivity of PEM	0.21
$\lambda_{cgdl}$ (W/(m·K))	Thermal conductivity of CGDL	1.6
$\lambda_{ccc}$ (W/(m·K))	Thermal conductivity of CCC	16
$D_{m,mem}^{eff}$ (m^2^/s)	Diffusion coefficient of PEM in methanol	4.9 × 10^−10^exp(2436 × (1/333 − 1/T))
$D_{m,acc}^{eff}$ (m^2^/s)	Diffusion coefficient of ACC in methanol	2.8 × 10^−9^
$D_{m,agdl}^{eff}$ (m^2^/s)	Diffusion coefficient of AGDL in methanol	2.8 × 10^−9^ε
$D_{O_{2},ccc}^{eff}$ (m^2^/s)	Diffusion coefficient of CCC in methanol	0.25 × 10^−4^
$D_{H_{2}O,air}^{eff}$ (m^2^/s)	Diffusion coefficient of water vapor in air	6 × 10^−4^
$C_{ref}^{O_{2}}$ (mol/m^3^)	Oxygen reference concentration	$0.21P_{0}/\left(RT_{amb}\right)$
$j_{a}$ (A/m^3^)	Anode reference exchange current density	11,000
$j_{c}$ (A/m^3^)	Cathode reference exchange current density	11,000
$R_{cell}$ (Ω)	Internal resistance	0.00008
$E_{cell}^{0}$ (V)	Open circuit voltage	1.213
$\frac{\partial E}{\partial T}$ (V/K)	The change rate of electromotive force	−1.4 × 10^−4^

**Table 2:** Typical transfer coefficients used in the model.

$\mathrm{Anode}\;\mathrm{CTC}/\alpha_{a}$	$\mathrm{Cathode}\;\mathrm{CTC}/\alpha_{c}$	Literature
0.35	0.8	[21]
0.45	0.85	[24]
0.5	0.875	[25]

**Table 3:** Physical parameters.

Parameter/Symbol	Unit	Value
PCB constant pressure heat capacity/${Cp}_{b}$	J/(kg·K)	1470
PCB density/$\rho_{b}$	kg/m³	1190
PCB thermal conductivity/$K_{b}$	W/(m·K)	0.2
PCB heat transfer coefficient/$h_{b}$	W/(m^2^·K)	4
Constant voltage heat capacity of devices/${Cp}_{d}$	J/(kg·K)	502
Devices density/$\rho_{d}$	kg/m³	8700
Devices thermal conductivity/$K_{d}$	W/(m·K)	17
Devices heat transfer coefficient/$h_{d}$	W/(m^2^·K)	7

**Table 4:** Heat sources corresponding to different sizes of DMFCs.

Concentration (mol/L)	Current Density (mA/cm^2^)	Size (cm)	Heat Source (W/m^2^)
3	50	2	258.2
3	50	1.5	600
3	50	1	246.43
3	50	0.5	221.65

**Table 5:** Temperature of program calculation and simulation verification for optimized layout.

T	T1 (K)	T2 (K)	T3 (K)	T4 (K)	Ttot (K)
1c	311.703	312.181	312.089	311.802	1247.775
1v	312.17	312.12	312.13	312.07	1248.49
2c	321.277	315.078	316.472	316.538	1269.365
2v	321.52	315.29	316.55	316.50	1269.86
3c	319.275	329.091	320.617	319.854	1288.873
3v	320.20	328.59	320.06	319.41	1288.26

**Table 6:** Initial and optimized layout coordinates.

Coordinate	(x1, y1)	(x2, y2)	(x3, y3)	(x4, y4)	Ttot (K)
1i	−0.02, −0.02	0.02, 0.02	−0.01, 0.01	0.01, −0.01	1228.59
1o	−0.00367, −0.00688	0.00778, 0.00432	−0.00349, 0.00425	0.00788, −0.00680	1248.49
2i	0, 0	0.011, 0	−0.011, 0	0, −0.011	1266.02
2o	−0.01027, −0.00596	0.00075, 0.005	−0.01029, 0.00504	0.00076, −0.00601	1269.86
3i	−0.02, 0.005	0.01, −0.03	0.01, 0.005	−0.005, −0.01	1256.16
3o	−0.0114, −0.0004	0.0077, −0.0005	0.0078, 0.0130	0.0077, −0.0115	1288.26

## Nomenclature

**Abbreviation**	**Full Name**	**Unit**
*Tank*	Liquid storage cavity	
*ACC*	Anode current collector	
*AC*	Anodic channel	
*AGDL*	Anode gas diffusion layer	
*ACL*	Anode catalyst layer	
*PEM*	Proton exchange membrane	
*CCL*	Cathode catalyst layer	
*CGDL*	Cathode gas diffusion layer	
*CCC*	Cathode current collector	
*CC*	Cathode channel	
$N_{m}$	Methanol flux	mol/(m^2^·s)
$C_{m,tank}$	Methanol concentration in liquid storage cavity	mol/m^3^
$C_{m,acc}^{0}$	Anode plate surface concentration	mol/m^3^
$C_{m,acc}$	Anode plate concentration	mol/m^3^
$C_{m,agdl}$	Anode diffusion layer concentration	mol/m^3^
$i_{a}$	Anode current density	A/m^2^
$N_{cross}$	Methanol permeation flux	mol/(m^2^·s)
$\eta_{a}$	Anode overpotential	V
$T_{acl}$	Anode catalytic layer temperature	K
$C_{O_{2},ccc}^{0}$	Cathode plate surface concentration	mol/m^3^
$C_{O_{2},ccl}$	Cathode catalytic layer oxygen concentration	mol/m^3^
$C_{O_{2},ccc}$	Cathode plate oxygen concentration	mol/m^3^
$C_{O_{2},cgdl}$	Cathode diffusion layer oxygen concentration	mol/m^3^
$i_{c}$	Cathode current density	A/m^2^
$i_{p}$	Permeation current density	A/m^2^
$\eta_{c}$	Cathode overpotential	V
$T_{ccl}$	Cathode catalytic layer temperature	K
$q_{acl}$	Heat produced by anode catalyst layer	W/m^2^
$q_{ccl}$	Heat generated by cathode catalytic layer	W/m^2^
$q_{tot}$	Total heat flux	W/m^2^
$h_{H_{2}O}$	Mass transfer coefficient of water vapor	
$C_{H_{2}O,CCC}^{sat}$	Concentration of water vapor in cathode plate	mol/m^3^
$P_{H_{2}O}$	Saturated pressure of water in wet air	Pa
